# Supplementary material for: Actin-binding domain of Rng2 sparsely bound on F-actin strongly inhibits actin movement on myosin II
Source: Life Sci Alliance. 2022 Oct 26;6(1):e202201469. doi: 10.26508/lsa.202201469 (PMC9610768; doi:10.26508/lsa.202201469)
Supplement: Supplementary file 8 [file LSA-2022-01469_TableS1.docx]

**Table S1. Binding ratio of Rng2CHD needed to achieve various degrees of motility inhibition***

| Degree of inhibition | | 50% | 75% | 80% | 90% | 95% |
| --- | --- | --- | --- | --- | --- | --- |
| Rng2CHD | Based on *K_d_* | 1.3% | 3.3% | 3.6% | 4.8% | 7.7% |
| GFP-Rng2CHD** | Based on *K_d_* | 4.3% | 6.7% | 7.1% | 13% | 20% |
|  | GFP-fluorescence | nd | nd | 9.7% | 21% | nd |

*: Effects of Rng2CHD on motility of actin filaments on muscle HMM were assayed in several independent sets of experiments under different conditions. The data shown here were measured in the presence of 0.5 mM ATP, which is different from those shown in Figure 1.

**: The estimated binding ratios of GFP-Rng2CHD needed to achieve the same degree of inhibition was slightly lower when estimated from *K_d_* than directly measured from the fluorescence intensities. However, both methods of estimation yielded higher required binding ratios than Rng2CHD, suggesting that the fused GFP moiety interferes with the activity of Rng2CHD to inhibit the motility of actin filaments on muscle HMM. nd: not determined.
