# Supplementary material for: Actin-binding domain of Rng2 sparsely bound on F-actin strongly inhibits actin movement on myosin II
Source: Life Sci Alliance. 2022 Oct 26;6(1):e202201469. doi: 10.26508/lsa.202201469 (PMC9610768; doi:10.26508/lsa.202201469)
Supplement: Supplementary file 11 [file LSA-2022-01469_TableS2.docx]

**Table S2**. Half helical pitch (HHP) of 0.59 µM actin filaments incubated with different concentrations of Rng2CHD at the equilibrium state (*K_d_* of Rng2CHD = 0.92 µM). The value is a mean ± SD. The differences between the mean HHP of control actin filaments (0 µM Rng2CHD) and those incubated with different Rng2CHD concentrations were statistically significant (*, p ≦ 0.001, two independent populations *t*-test). The concentration of actin was 0.59 µM. For details, see Figure 4.

| Rng2CHD (µM) | HHP (mean ± SD) (nm) | Number of HHPs measured | *t*-test  *p*-value |
| --- | --- | --- | --- |
| 0.0 (control) | 36.4 ± 3.1 | 2225 |  |
| 0.020 | 35.5 ± 2.9 | 1196 | 4.9 x 10^-16^ |
| 0.25 | 35.0 ± 3.0 | 1026 | 4.4 x 10^-31^ |
| 0.85 | 34.1 ± 4.0 | 1265 | 1.7 x 10^-77^ |
| 2.6 | 34.2 ± 3.8 | 917 | 3.7 x 10^-62^ |
| 5.7 | 34.2 ± 4.0 | 945 | 1.5 x 10^-60^ |
